# Pulsed Electric Field: Fundamentals and Effects on the Structural and Techno-Functional Properties of Dairy and Plant Proteins

**DOI:** 10.3390/foods11111556

**Published:** 2022-05-25

**Authors:** Ahmed Taha, Federico Casanova, Povilas Šimonis, Voitech Stankevič, Mohamed A. E. Gomaa, Arūnas Stirkė

**Affiliations:** 1 Department of Functional Materials and Electronics, Center for Physical Sciences and Technology, Saulėtekio al. 3, LT-10257 Vilnius, Lithuania; ahmed-taha@alexu.edu.eg (A.T.); povilas.simonis@ftmc.lt (P.Š.); voitech.stankevic@ftmc.lt (V.S.); 2 Department of Food Science, Faculty of Agriculture (Saba Basha), Alexandria University, Alexandria 21531, Egypt; m.gomaa@alexu.edu.eg; 3 Food Production Engineering, National Food Institute, Technical University of Denmark, 2800 Kongens Lyngby, Denmark; 4 Micro and Nanodevices Laboratory, Institute of Solid State Physics, University of Latvia, Kengaraga Str. 8, LV-1063 Riga, Latvia

**Keywords:** pulsed electric field, pulse generation, milk proteins, plant proteins, functional properties, protein structure

## Abstract

Dairy and plant-based proteins are widely utilized in various food applications. Several techniques have been employed to improve the techno-functional properties of these proteins. Among them, pulsed electric field (PEF) technology has recently attracted considerable attention as a green technology to enhance the functional properties of food proteins. In this review, we briefly explain the fundamentals of PEF devices, their components, and pulse generation and discuss the impacts of PEF treatment on the structure of dairy and plant proteins. In addition, we cover the PEF-induced changes in the techno-functional properties of proteins (including solubility, gelling, emulsifying, and foaming properties). In this work, we also discuss the main challenges and the possible future trends of PEF applications in the food proteins industry. PEF treatments at high strengths could change the structure of proteins. The PEF treatment conditions markedly affect the treatment results with respect to proteins’ structure and techno-functional properties. Moreover, increasing the electric field strength could enhance the emulsifying properties of proteins and protein-polysaccharide complexes. However, more research and academia–industry collaboration are recommended to build highly effective PEF devices with controlled processing conditions.

## 1. Introduction

Food proteins play vital roles in human nutrition, food production, and nutraceutical industries. Dairy proteins have excellent functional properties and high nutritional values [1,2]. The dairy industry also significantly contributes to the world economy, particularly in the European Union (EU). For example, according to the milk market observatory of the European Commission, the EU exported more than 500 thousand tons of whey powders in 2020 [3]. As a raw material in many food products, dairy proteins are required to possess excellent functional properties, including high solubility and improved emulsifying, foaming, and gelling properties [4]. To improve the functional properties of dairy proteins, several changes in the structural and conformational state of such proteins are needed. For instance, the solubility of proteins depends mainly on the hydrophilic and hydrophobic residues on the proteins’ surfaces, as well as the content of hydrogen bonds. Moreover, the gelling properties could be altered if the contents of sulfhydryl (SH) groups and disulfide bonds are changed. Additionally, the emulsifying properties are closely related to the surface activity of proteins [5]. Plant proteins are increasingly being utilized as alternative sources of animal proteins due to their importance in developing sustainable food systems [6,7]. Moreover, plant proteins have been used as emulsifiers, foam stabilizers, and for other applications in the food industry due to their affordable price and amphiphilic structures [8,9]. The weak electrostatic repulsion, low solubility, and high molecular weight of proteins can limit the applications of these proteins [10]. Therefore, emerging green technologies are needed to alter the structural and techno-functional properties of plant and dairy proteins with minimal effects on the nutritional value and flavor of these proteins and their products. 

Electric field devices are classified into two major categories based on the electric field strength. Pulsed electric field (PEF) devices have electric field strengths of 20–100 kV/cm, whereas moderate electric field (MEF) devices have electric field strengths of <1000 V/cm [11]. PEF, as an emerging ecofriendly technology, has been used in the food industry for the inactivation of enzymes and microorganisms [12,13,14,15,16]. During PEF treatment, pulses of high-voltage electric fields for a short time (from nanoseconds to milliseconds) were applied to the material between two electrodes [17,18]. PEF technology offers several advantages over traditional pasteurization methods in the food industry, such as shelf-life extension, nutrient retention, quality preservation, and cost effectiveness [19,20]. Therefore, PEF is being adopted increasing rapidly in many industrial sectors, including for the processing of plant and dairy products [13,21]. Some studies have shown that PEF could be used to alter the structural and techno-functional properties of food proteins [5,22,23,24,25,26,27,28,29,30,31,32,33,34].

The main mechanism behind the effects of electric fields on proteins is not very clear. However, some researchers have proposed that polar groups of proteins absorb energy during PEF treatment and generate free radicals. The produced free radicals can affect intramolecular interactions within protein molecules, including hydrophobic and electrostatic interactions, disulfide bridges, hydrogen bonds, salt bridges, and Van der Waals forces [5]. Moreover, PEF treatments could change the apparent charge of proteins due to the alteration of their ionic interactions [4,35]. Therefore, the structural and functional properties of proteins can be altered. Several review articles have discussed the effects and food applications of PEF technology [4,5,13,14,18,20,26,36,37,38,39,40,41,42,43,44,45,46,47]. However, there is a lack of systematic reviews discussing PEF fundamentals and their applications in the food industry. Moreover, to the best of our knowledge, few studies have been conducted on the effects of PEF on the structure and techno-functional properties of food proteins; thus, systematic reviews discussing this point are scarce. Therefore, a review of the fundamentals of PEF and the effects of PEF treatments on the structural and techno-functional properties of food proteins is needed. Accordingly, with this review, we aim to (1) briefly compare PEF with other processing techniques; (2) discuss the theory and fundamentals of PEF technology; and (3) discuss the effects of PEF treatments on the structural and techno-functional properties (including solubility, gelling, emulsifying, and foaming properties) of dairy and plant proteins. This review could guide both researchers and industry leaders to develop new applications of PEF as a green and sustainable technology in the food industry.

## 2. PEF vs. Other Processing Technologies

Besides PEF, some other emerging technologies have been used to alter the structure and techno-functional properties of food proteins, such as ultrasound [48,49,50,51], high-pressure processing [52,53,54,55], microwave treatment [56,57,58,59], and cold plasma processing [60,61,62]. The main mechanisms, processing parameters, and effects of these technologies on proteins are summarized in Table 1. However, there is currently a growing interest in PEF because of its sustainable approach and wide range of applications in the food and biotechnology sectors [63]. For example, PEF can be used for:

- Microbial inactivation [64];
- Enhancing the quality of freeze-dried fruits [65] and improving the quality of potato chip products [66];
- Improving drying and extraction kinetics [67];
- Winemaking, biogas production, and extraction of protein from algae [63]; and
- Improving the functional properties of proteins [5].

Moreover, PEF offers many advantages over other techniques [47], including:

- Short processing times;
- Waste-free process;
- Low energy consumption;
- Environmentally-friendly technique;
- Better retention of nutrients, flavors, and colors; and
- Suitability for processing heat-sensitive foods.

The main disadvantage of PEF technology is the existence of bubbles during treatment, which could result in operational problems and non-uniform treatment. Additionally, commercial PEF units not widely available in many regions worldwide [47].

**Table 1:** Brief comparison of different emerging processing techniques and their effects on protein structure and techno-functional properties.

Processing Technology	Processing Parameters	Mechanism	Effects on Protein Structure	Effects on Proteins’ Techno-Functional Properties
PEF	Pulse-wave shape, pulse duration, electric field strength, frequency, temperature, and treatment duration [68].	Polarization of protein molecules and release of free radicals can induce changes in protein structures and functionalities [4].	Depends on the electric field strength and the type of proteins. Main changes occurred in the secondary structure and exposure of hydrophobic groups to the surface of protein molecules [33].	Different waveforms and protein types can have different effects on protein solubility. Emulsifying and foaming properties improved [69].
Ultrasound	Amplitude, frequency, acoustic energy, intensity, energy density (J/mL), time, and temperature [70].	Acoustic cavitation (the formation and collapse of air bubbles) induces chemical reactions and physical effects, which influence the structure and techno-functional properties of proteins [71].	Changes in the secondary and tertiary structures. Increases in surface hydrophobicity and free sulfhydryl groups [51]	Ultrasound improved the emulsifying and gelling properties of proteins [72].
High pressure processing	Pressure, temperature, and time [53]	Protein unfolding can occur due to the penetration of water into the protein matrix [38].	Depends on the applied conditions and protein system. Mainly protein denaturation and aggregation occurred [73].	Depends on the applied pressure. Emulsifying and foaming capabilities enhanced. Solubility of proteins improved [74].
Microwave	Power, frequency, time, and temperature [56].	At the molecular level, exposed proteins interact with electromagnetic energy. Then, heat is generated from the electromagnetic energy through the motion of molecules during treatment [71].	Changes in the secondary structure. Protein aggregation [75].	Gelling properties improved [76].
Cold-plasma processing	Voltage, frequency, time, and temperature [77].	Several high-energy radicals, such as nitric oxide, atomic oxygen, superoxide, and hydroxyl radicals to break the covalent bonds and promote several chemical reactions [78].	The high-energy reaction could break peptide bonds and oxidize the side chains of amino acids. They may also facilitate the formation of protein–protein interactions. Changes in the secondary structures were observed [61].	Water- and oil-holding capacities enhanced, reflecting the improvement of emulsifying and gelling properties of proteins [62].

## 3. Fundamentals of PEF Technology: Device Components and Pulse Generation

An electric circuit simply means a closed-loop that carries electricity. The electric current (*I*) is the flow of electrons in the circuit, measured in amperes (A) and can be calculated according to Equation (1). The voltage (*V*, in volts) is the electric pressure or source that causes the flow of the current. The resistance (*R*, in ohms) in the electric circuit opposes the current flow [79].

(1) $$I=\frac{V}{R}$$

Contemporary PEF is based on a direct application of power pulses to the food material placed between two electrodes for micro- to nanoseconds at an intensity range of 10–80 kV/cm [80]. The PEF processing time can be calculated by multiplying the effective pulse duration by the number of pulses. The magnitude and time course of PEF are controlled by a voltage generator and electrode geometry.

**Figure 1:**
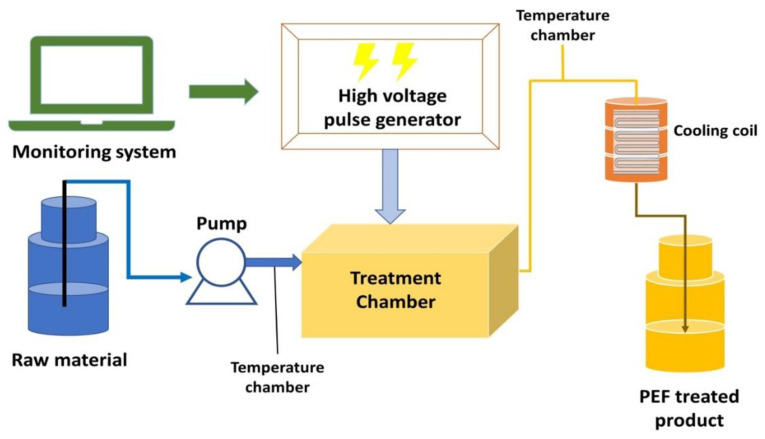
Diagram of a possible continuous PEF device used to treat food samples.

PEF devices basically consist of a treatment chamber with a suitable cuvette, a high-voltage pulse generator, and necessary controlling and monitoring devices. Figure 1 shows a diagram of a continuous PEF device used to treat food samples. The electric field depends on the applied electric voltage, the distance between the two electrodes, and pulse width and waveform (Equation (2)). *E* represents the electric field strength (V/m), *u*(*t*) represents the applied voltage over time (*V*), and *d* represents the distance between electrodes (m) [81].

(2) E(t)=1d∗∫0tu(t) dt

**Figure 2:**
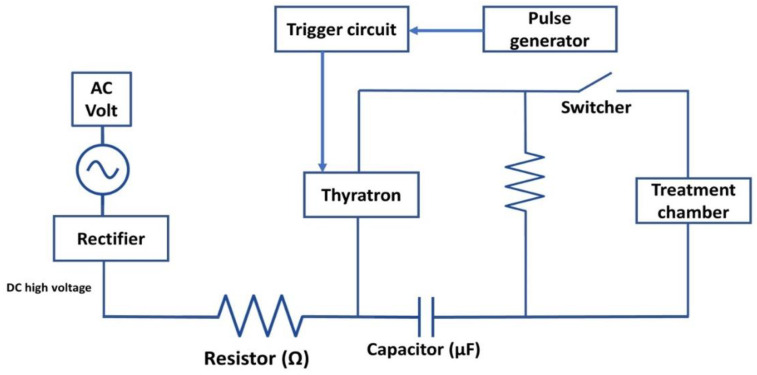
Schematic diagram of an electric circuit in a PEF device used for food processing.

Many types of circuits (pulse generators) in PEF devices have different circuit components to fulfill the required functions. Figure 2 is a schematic diagram that represents an example of an electric circuit in a PEF device used for food processing. Electrical pulses are acquired by charging a capacitor, and the discharging of the capacitor is controlled by a trigger or switcher that controls the decay in an electronic circuit [82]. Table 2 summarizes the components of PEF systems and their functions. As shown in Table 2, the electric circuit of PEF devices employed for food processing has several electric elements. A high-voltage pulse generator is used to charge the capacitors and can be a direct current (DC) or alternating current (AC) switched to DC using a rectifier. The high-voltage pulse generator is also used to discharge the high voltage from capacitors in the form of a pulse with a specific pulse shape and width through a pulse-forming network (PFN) (Figure 2).

A capacitor is an electrical device used to store energy that used to generate electric pulses in an electric circuit (PEF systems). Several types of capacitors are available in the market, such as electrolytic, ceramic, paper, film, mica, and non-polarized capacitors [86]. Electrochemical capacitors (ECs), also known as supercapacitors or electrochemical double-layer capacitors (EDLC), have recently been used in many electronic applications on a large scale [84]. Generally, capacitors consist of two parallel conductive (metal) electrodes isolated using non-conducting materials (dielectrics), such as ceramic, waxed paper, plastic, mica, or a liquid gel, as utilized in electrolytic capacitors. Due to the presence of dielectric materials between two conductive materials, the direct current cannot flow through the capacitor. Thus, a voltage is stored in the conductive metal plates as an electrical charge [84]. Several factors determine the power needed to charge the capacitor, such as the size and number of capacitors, as well as the resistance of the charging resistor [87]. It has been concluded that a larger capacitor requires more power and time to be charged than a smaller one. The capacitance, *C*_0_ (*F*), of a capacitor can be calculated with Equation (3), where *R* (Ω) is the resistance, *A* (m^2^) is the area of the electrode surface, *σ* (S/m) is the conductivity of the food, *τ* (s) is the pulse duration, and *d* (m) is the distance between electrodes [80].

(3) $$C_{0}=\frac{\tau }{R}=\frac{\tau \sigma A}{d}$$

The energy stored (*Q*) in a capacitor is calculated based on the values of capacitance (*C*_0_) and charge voltage (*V*), as shown in Equation (4) [80].

(4) $$Q=0.5\;C_{0}\;V^{2}$$

**Figure 3:**
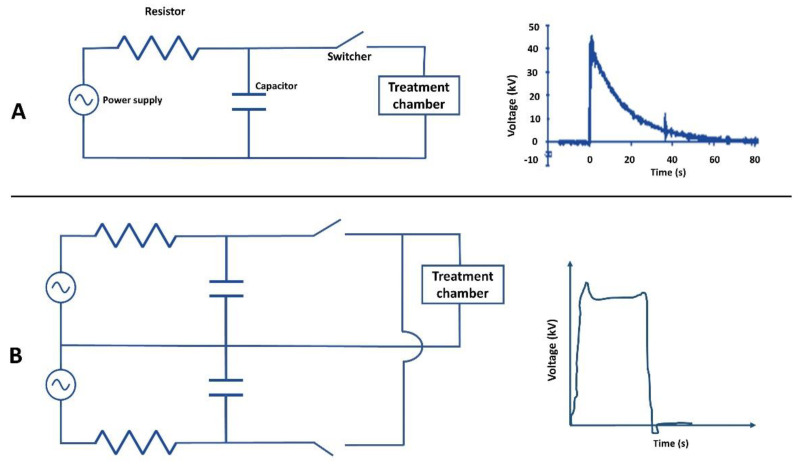
The two main types of electrical circuits and their possible pulses shapes: (**A**) simple resistance–capacitance (*RC*) circuit and its exponentially decaying pulse shape; (**B**) complex electric circuit and its square pulse shape.

The switching device (switcher) is a critical device for the efficiency of PEF systems. It is needed to connect or disconnect the electric current and discharge the stored energy in a capacitor through the PFN. There are two main types of switchers: ON (semi-controlled) and ON/OFF (fully controlled) switchers. The suitable switcher in a circuit is selected based on its repetition rate and potential to operate at high voltage. Capacitors should be fully discharged to turn the switcher off in semi-controlled switches (including thyratron, trigatron, gas spark gap, and ignitron). These switches can control high voltages at a lower cost. However, low repetition rate and short life are the main disadvantages of such switches. Fully controlled switches (including symmetrical gate commutated thyristors (SGCT), insulated gate bipolar transistors (IGBT), and the gate turn-off (GTO) thyristors) can control the pulse generation process and can be switched on and off with full or partial discharge of the capacitors. The development of fully controlled semiconductor switches increased life spans and improved switch performance [20,80]. The relative electrical value of each component of PFN systems influences the pulse shape. For instance, an exponentially decaying pulse shape is formed in a simple resistance–capacitance (*RC*) circuit. On the other hand, complex PFN systems can produce instantaneously reversal, as well as bipolar and square pulses (Figure 3). To generate exponential decay pules, the generation of an exponential decay pulse only needs semi-controlled (ON) switchers in which the capacitor is completely discharged. Square wave pulses can be generated by the partial discharge of a capacitor with fully controlled (ON/OFF) switchers [85].

Resistors are one of the main components of electric circuits. They are used to regulate the current flow and force voltage reduction. The theory of electric resistance is similar to the water flow in pipes; the resistor can be considered a thin pipe (wire in the case of an electric circuit) that reduces the water flow [83]. In reducing the current flow, the electrical energy is absorbed by the resistor and dissipated as heat. Capacitors are classified based on their production materials: wire-wound, film, or cermet (made of metal or metal-oxide); carbon composition; and semiconductor capacitors. Ohm’s law of resistance specifies the relationship between the resistance (*R*), voltage (*V*), and current (*I*) (Equation (1)); an ohm (Ω) equals a volt per ampere [83,88].

**Figure 4:**
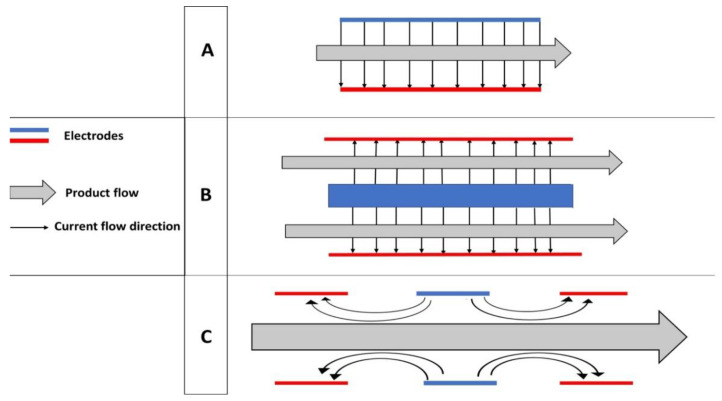
Schematic diagrams of the three main types of treatment chambers in PEF devices. (**A**) parallel plate chambers; (**B**) coaxial plate chambers; (**C**) colinear plate chambers.

Various treatment chambers are designed to expose food samples to the electric field. Static chambers are used in batch processing and laboratories, whereas continuous chambers can meet the requirements of industrial-scale applications. Batch chambers offer several advantages at a laboratory scale, such as treating small-volume samples, efficiently controlling temperature by cooling the electrodes, and slowing the repetition rates. However, continuous chambers are essential to reach high-volume capacity; they are also easily integrated into existing food processing lines [81,85]. The material used for building treatment chambers should be washable or autoclavable. Currently, three main types of treatment chambers are designed based on the arrangement of electrodes in different geometric configurations, including parallel plates, as well as coaxial and colinear chambers [41,85]. Parallel and coaxial plates are commonly used for batch processing, whereas colinear chambers are utilized in continuous processing devices (Figure 4).

Recently, several companies have developed emerging PEF systems for industrial applications. Current large-scale PEF devices are based on Marx generators or transformers, and electric pulses are applied continuously. In Marx generators, a stack of capacitors is used, charged in parallel, and discharged in a sequence, providing a high-power conversion rate. For transformers, a pulse transformer is used with a low-voltage switch. Most PEF units have an average power ranging from 20 to 400 kW [89]. Treatment chambers are designed based on the scope of application. Two major types of treatment chamber are belt systems, which are used for processing of solid products, such as potatoes or seafood, and pipe systems are used for processing of liquid products [90].

The outcome of PEF treatment is influenced by many product and process factors. Product factors include chemical composition, pH, rheological properties, temperature, and electrical conductivity. Process parameters include electric field strength, pulse number, pulse frequency, the shape of the pulse wave, pulse width, type of treatment chamber, flow conditions, and flow rate [91]. It is worth mentioning that it is difficult to compare the data from different research groups due to many factors that affect PEF treatment results, which will be discussed in this review. In the following sections, we will discuss the effects of PEF on the structural and techno-functional properties of milk proteins.

## 4. Effects of PEF on the Structure of Dairy and Plant Proteins

**Figure 5:**
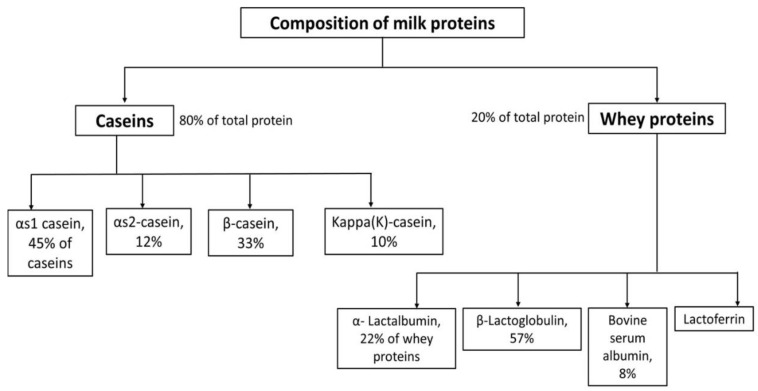
Flow chart of the composition of bovine milk (Data collected from Abd El-Salam et al. [92] and Onwulata et al. [93]).

As shown in Figure 5, caseins consist of four major subunits, including αs1, αs2, β, and kappa caseins. Whey proteins have several subunits, including β-lactoglobulin (β-LG), α-lactalbumin (α-LA), bovine serum albumin (BSA), lactoferrin, and traces of some other components, such as immunoglobulins and glycomacropeptide [92,93]. In general, upon thermal treatment of milk proteins, proteins unfold because of covalent bonds breaking, and sulfhydryl (-SH) groups are exposed to the protein surface; then, aggregates are produced due to the formation of disulfide bonds.

Moreover, as free thiol groups are not available in α-LA, it is less sensitive to thermal treatment than β-LG [4]. Studies have shown that PEF can change the structure of dairy proteins, especially at higher electric strengths at a wide range of temperatures [18]. The energy generated by PEF devices could expose amino acid and/or free-SH groups to the protein molecules’ surface. Moreover, non-covalent interactions, such as hydrophobic and hydrogen bonds, may be disrupted [4]. Furthermore, it was found that PEF can change the charge density around amino acids (at the -COOH and -NH3^+^ moieties), influencing the catalytic activity of peptides [94]. Whey proteins have recently attracted attention due to their nutritional benefits and industrial applications.

As summarized in Table 3, the available results in the literature about PEF effects on the structure of whey proteins are somehow contradictory. Sui et al. [95] investigated the effects of PEF and heat treatments (30–35 kV/cm, 19.2–211 µs, 30–75 °C) on the physicochemical and functional properties of whey protein isolates (WPI). They concluded that PEF treatment did not influence protein unfolding, surface hydrophobicity, of free-SH group content [95]. Using a different treatment chamber with a different distance between electrodes, Xiang et al. [22] found that PEF increased the surface hydrophobicity and the extrinsic fluorescence intensity of WPI. Similarly, Perez et al. [31] noticed that PEF treatment (12.5 kV/cm) with up to 10 pulses changed the native structure of β-LG and induced protein aggregation. The differences between the results may occur due to the use of various experimental conditions, such as treatment chamber, electric field intensity, frequency, and temperature [20]. Bovine lactoferrin was treated using PEF at different temperatures (30–70 °C) and compared with non-PEF-treated samples at the same temperatures [24]. The results showed that the lactoferrin concentration was not changed by the PEF treatment (35 kV/cm, 19.2 µs, 30–70 °C). Moreover, SDS-PAGE results indicated no significant difference in the gel profile of PEF and non-PEF-treated lactoferrin. The surface hydrophobicity increased with increased temperature. There were no significant differences in surface hydrophobicity values between PEF- and non-PEF-treated lactoferrin [24]. Bekard et al. [96] studied the effects of a low-intensity electric field on the conformational state of BSA using circular dichroism (CD) spectroscopy. They concluded that a low-intensity electric field (500 V/m, 3 h at 22.7–24.2 °C) changed the tertiary structure of BSA, probably due to perturbation in the hydrogen bonds that stabilized the native structure of BSA [96]. Sharma et al. [97] preheated milk samples to 55 °C for 24 s and then applied PEF at 20–26 kV/cm for 34 µs. The results indicated that the surface hydrophobicity of milk proteins considerably increased with increased electric field intensity. Thermal treatment at 30–55 °C can dissociate β-LG dimers into monomers [98]. Thermal pre-treatment associated with PEF might facilitate the dissociation of β-LG dimers of milk samples and expose hydrophobic groups and free-SH groups to the protein molecules’ surfaces [97]. Rodrigues et al. [99] compared conventional heat treatment with moderate electric field (MEF, 20–80 V/cm) heating at 50–90 °C. They found that with 70 °C and 80 °C treatments, moderate electric field treatment exhibited higher content of α-helix and random coils and lower content of β-sheet compared to conventional heat treatment at the same temperature. These structural changes probably occurred due to the effects of both heat treatment and electric field on the conformational state of β-LG.

Caseins are the major proteins in milk (80% of total milk protein) and one of the main protein sources in human nutrition. Studies on the effects of PEF on the structure of caseins are scarce. Subaşı et al. [33] studied the impact of MEF (230 V/cm) on the structural changes of sodium caseinate compared to sunflower protein. FTIR data revealed that MEF can change the secondary structure of sodium caseinate and unfold the protein molecules. This is probably because MEF treatment can polarize the surface of protein molecules, facilitating the exposure of hydrophobic regions to the surface of protein molecules [31,33].

The mechanism of PEF effects on milk protein structures can be proposed based on the available information in the literature. Generally, PEF treatments at low electric field intensities have no apparent effects on the structure of milk proteins. In contrast, PEF treatments at high electric field intensities can considerably change protein structures, especially in whey proteins.

**Figure 6:**
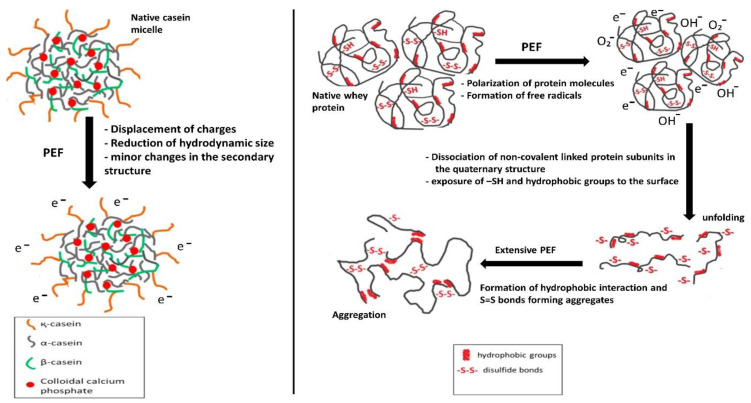
Mechanism of PEF effects on the structural properties of milk proteins (caseins and whey proteins); adapted with permission (License number: 5001830472118) from [4]. Copyright (2019) Elsevier.

As summarized in Figure 6, some polar groups of milk proteins absorb energy and produce free radicals when exposed to intensive electric fields. These free radicals can disrupt the several interactions among protein molecules, including disulfide and hydrogen bonds, as well as hydrophobic, electrostatic, and Van der Waals interactions. Moreover, the electric field can affect the strong dipole moment of the polypeptide chains, increasing the dielectric constant of proteins. These changes may facilitate the unfolding of protein molecules and the exposure of hydrophobic and -SH groups to the surface of protein molecules. Increasing the duration of PEF treatment could result in the formation of aggregates, as covalent and hydrophobic interaction may occur to crosslink unfolded protein molecules [4,5,31]. It is worth mentioning that an increase in temperature during PEF treatment could facilitate the denaturation of protein molecules. Thus, further study of the effects of PEF on protein structures under controlled temperatures is recommended.

As shown in Table 4, PEF treatment changed the structures of plant proteins. The secondary structure of soy protein isolate (SPI) changed after PEF treatment at 30–50 kV/cm. PEF caused denaturation and aggregation to SPI, probably due to the formation of hydrophobic interactions and S–S bonds [29,101]. Exposure of sunflower protein to moderate electric field strength (150 V for 20 s at a temperature < 45 °C) resulted in secondary and tertiary structural changes. PEF treatment broke the hydrophobic bonds and facilitated the crosslinking of amino acid side chains [33]. Similar results were also reported with pea and canola proteins [69,102]. Generally, PEF treatment is able to alter the structure of plant proteins. These changes could also affect the techno-functional properties of such proteins.

## 5. Effects of PEF on the Techno-Functional Properties of Dairy and Plant Proteins

The functionality of milk proteins is determined by physicochemical properties that affect the behavior of proteins during their utilization in food systems [103]. The modifications of protein structures can alter their functional properties [10]. Techno-functional properties, including solubility, gelling, emulsifying, and foaming properties, are of considerable interest in the food industry [104]. Therefore, in this section, we will discuss the effects of electric field treatment on the techno-functional properties of milk proteins. Table 5 presents the main studies on the effects of PEF on the techno-functional properties of dairy proteins, whereas Table 6 summarizes studies on the effects of PEF on the techno-functional properties of plant proteins.

### 5.1. Protein Solubility

Protein solubility is commonly determined by measuring the concentration of soluble proteins after the centrifugation of protein samples and relating it to the total protein concentration before centrifugation [108]. Protein solubility is influenced by several intrinsic factors, such as amino acid composition, protein molecular weight, the content of hydrophilic and hydrophobic groups on proteins molecules’ surfaces, and the content of hydrogen bonds [53,109]. Several extrinsic factors can also affect the protein solubility, including temperature, ionic strength, pH, and the presence of solvents [110]. Protein solubility is important for several protein applications, such as emulsions and foams. Therefore, it is recommended to use highly soluble proteins to form well-dispersed colloidal systems [111]. The effects of electric field treatments on the solubility of several proteins were investigated. There was a decrease in the solubility of pea (from 23.2 to 17.2%), rice (from 16.4 to 9.2%), and gluten (from 25 to 22.4%) concentrates after treatment with moderate electric field strength (1.65 kV/cm, square pulse system) [112]. Similarly, the content of soluble egg white proteins decreased (7.84%) after PEF treatment using a PEF system with square-wave pulses (at 25 kV/cm) [113]. The authors also observed that the average particle size of egg white proteins increased (36.9%) after PEF treatment. PEF unfolded protein molecules and formed insoluble protein molecules. Moreover, intermolecular interactions, such as S-S bonds could occur, resulting in reduced protein solubility. However, with soy protein isolates, Li et al. [29] found that PEF treatment of up to 30 kV/cm using a PEF system with bipolar waveforms improved solubility, whereas PEF at strengths higher than 30 kV/cm resulted in a slight decrease in protein solubility. Additionally, PEF treatment (35 kV/cm for 8 μs) increased the solubility (50.07%) of canola protein compared to that of control samples (43.25%) [69]. Therefore, we conclude that different waveforms and protein types can affect protein solubility differently. However, there is a lack of available knowledge about factors behind the desired solubility of milk proteins after PEF treatment, probably due to the confirmed higher solubility of milk proteins.

### 5.2. Gelling Properties

**Figure 7:**
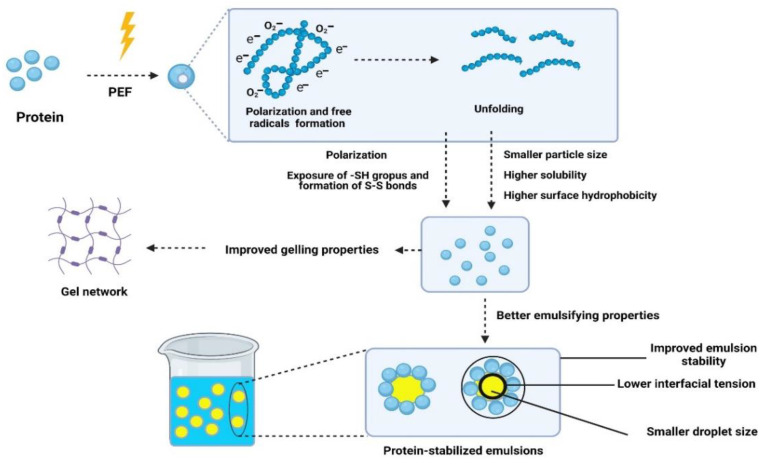
Proposed mechanism of PEF effects on the emulsifying and gelling properties of proteins. This figure was created with BioRender.com, (accessed on 18 May 2022) with publication permission.

The gelling properties of proteins are closely associated with the content of -SH groups and disulfide bonds. In the dairy industry, the gelation of milk proteins is an essential factor influencing the quality of many dairy products, including cheese, yogurt, and dairy-based desserts [114]. Perez et al. [31] found that PEF improved the gelling rate of β-lactoglobulin (at 72 °C) when samples were exposed to fewer than six pulses. Yu et al. [105] studied the effects of PEF (20 and 30 kV/cm) at different outlet temperatures on the rennet coagulation characteristics of raw milk. They found that PEF (at 20 °C)-treated milk had higher curd firmness than pasteurized milk samples. Moreover, PEF-treated milk samples had a lower rennet coagulation time (RCT) than pasteurized milk samples. It is known that lower RCT values result in better gelling properties [105]. Jin et al. [107] concluded that the gelling properties of WPI increased when treated at 35 kV/cm but decreased after PEF treatment at 45 kV/cm. As proposed in Figure 7, during PEF treatment, the unfolding of milk proteins and the exposure of -SH groups, followed by the formation of S-S bonds, could be the reason behind the improved gelling properties of milk. Another reason for the improved gelling properties reported in these studies could be the polarization of protein molecules during treatment. Polarized molecules can attract each other through electrostatic forces [31]. However, Sui et al. [24] found that PEF (30 kV/cm)-treated WPI showed lower gel strength than untreated samples; increasing the PEF duration decreased the gel strength of WPI samples. Rodrigues et al. [106] concluded that conventional heat-treated WPI samples had higher gel strength than those subjected to moderate electric field treatment (15–22 V/cm). At pH 7, electrostatic repulsion among protein molecules may reduce the size of protein aggregates [115]. Moreover, applying an electric field could destroy some of the non-covalent bonds between proteins [106]. The water-holding capacity of PEF-treated canola protein increased at lower electric field strength (25 kV/cm) and decreased at higher electric field strength (35 kV/cm) [69]. For pea protein isolate, lower electric field treatment resulted in cohesive, more elastic, and weaker gels with higher water-holding capacity [102]. The inconsistency of gelling properties reported in different studies could be due to the use of different PEF conditions, such as voltage, the shape of the pulse wave, and the type of treatment chamber used.

### 5.3. Emulsifying and Foaming Properties

The stability of emulsions is vital for improving the shelf life of emulsion-based food products, such as mayonnaise, ice cream, butter, milk, and margarine. Therefore, several emulsifiers are used to reduce interfacial tension, improving the stability of emulsions [70]. Among them, proteins are widely used as natural emulsifiers due to their surface-active properties [116,117]. Several processing technologies, such as high-pressure treatment [53], ultrasound [70,118], cold plasma treatment [61], and microwaves [56], have been used to improve the emulsifying properties of proteins. Studies on the effects of PEF on the emulsifying foaming properties of milk proteins are scarce. Sui et al. [95] compared the effects of heat treatment and PEF on the emulsifying properties of WPI. They observed that emulsions stabilized by PEF-treated (30 kV/cm) and heat-treated (72 °C for 15 s) samples had similar droplet sizes (~4 μm), whereas emulsions stabilized by WPI heated for 10 min had significantly larger droplet sizes (18.3 μm). Sun et al. [119] studied the effects of PEF treatment (15 and 30 kV/cm) on the emulsifying properties of a WPI–dextran mixture. They found that the PEF-treated mixture had a higher emulsifying activity index (EAI) than the untreated mixture [119]. PEF could facilitate the glycosylation reaction between WPI and dextran. The combination of protein and polysaccharides was confirmed to improve the stability of emulsions. This could be because the hydrophobic regions of proteins can be adsorbed at the surface of oil droplets, and the hydrophilic part of polysaccharides can be oriented towards the water phase, preventing the coalescence of oil droplets through steric stabilization [70]. Zhang et al. [69] found that PEF pre-treatment of canola seeds prior oil and protein extraction improved the emulsifying and foaming properties of the resulting canola proteins. PEF could improve the solubility of plant proteins and promote the exposure of their hydrophobic groups to the surface, thus improving their emulsifying and foaming properties. More studies are needed to understand the effects of PEF treatment on the emulsifying and foaming properties of plant and milk proteins. The changes in the protein structures induced by PEF treatment could improve the techno-functional properties of proteins. As proposed in Figure 7, PEF could polarize and unfold protein molecules, exposing the hydrophobic groups to the surface of molecules [5]. Additionally, PEF can increase solubility and reduce the particle size of protein molecules at a certain electric field strength. These changes could reduce the interfacial tension at the oil/water interface, improve the emulsifying properties of food proteins, and enhance the stability of protein-stabilized emulsions [11]. However, there is still a lack of detailed information on the mechanism of action of PEF and its effects on protein functionality due to the limited number of studies conducted in this area. Thus, more fundamental research at the molecular scale is required to establish a clear mechanism of PEF effects on protein functionality.

## 6. Conclusions and Future Perspectives

Pulsed electric field is a promising green technology that can be utilized in many food applications. With an increase in sustainable development needs, the utilization of PEF in the food industry is expected to increase in the coming years. The conclusions of the available studies that investigated the effects of PEF on the structure and techno-functional properties of milk and plant proteins can be summarized in the following points:

- In general, PEF treatment at low electric strength (<10 kV/cm) cannot change the structure of proteins;
- PEF treatment conditions, such as electric strength, pulse shape, pulse duration, and the type of treatment chamber, have a significant impact on the effects of PEF on the structure and techno-functional properties of proteins’
- The effects of PEF on structure and techno-functional properties are vary from one protein type to another.

As a limited number of studies have been conducted to investigate the effects of PEF on food proteins, several aspects need to be investigated in the future. The impact of different electric field strengths on the structure and techno-functional properties of proteins must be studied to define the optimum PEF conditions to improve the techno-functional properties of such proteins. Investigation of the impacts of using nanosecond PEF treatment on the structure and techno-functional properties of food proteins is recommended. Moreover, PEF, as a promising green technology, can be introduced at a large scale to produce highly effective emulsifiers. Research could be conducted to determine the possibility of using PEF technology as an emulsification technique to produce food-based stable emulsions. The main challenge of PEF applications is that many factors (such as PEF device parameters and external factors, such as conductivity, pH, and concentration of treated solutions) can affect the treatment results. Consequently, studies focusing on thermal, chemical, and biophysical components of PEF effects on protein structures should be conducted until clear mechanisms are elucidated. It is also extremely important that authors provide all the necessary details about treatment conditions so the analog studies can be implemented, and results can be compared between those studies. We recommend referring to the guidelines and recommendations proposed by Cemazar et al. [68] for reporting on PEF applications. Moreover, collaborations between the food industry and academic institutions are needed to design and build more effective and energy-efficient PEF devices with controlled treatment conditions.

## Figures and Tables

**Table 2:** Functions of main PEF devices components.

Component	Description and Function(s)	References
High-voltage pulse generator	To generate high-voltage direct current (DC) at a specific intensity by a power supply. To discharge high voltage in the form of pulses with specific shapes and widths through a pulse-forming network (PFN).	[80]
Resistors	Delay the current flow and impose a voltage reduction.	[83]
Capacitors	Energy (voltage) storage.	[84]
Switchers	Connect or disconnect the electric current and control the discharge of the stored energy.	[20]
Treatment chambers	Specific containers are used to carry food samples during exposure to PEF.	[81,85]

**Table 3:** Effects of pulsed electric field (PEF) on the structure of dairy proteins.

Dairy Protein	PEF Conditions	Structural Changes	References
Whey protein	35.5 kV/cm for 300 or 1000 µs, pulse duration of 7 µs, and pulse repetition rate set at 111 Hz.	Significant differences in the concentration of α-LA, β-LG, and serum albumin between PEF-treated samples for 300 µs and 1000 µs.	[100]
Whey protein isolate (WPI)	12, 16, and 20 kV/cm; number of pulses (10, 20, and 30)	More hydrophobic groups exposed. Partial denaturation of WPI fractions.	[22]
WPI	30–35 kV/cm, 19.2–211 µs, 30–75 °C, flow rate of 60 mL/min	No obvious changes in the gel pattern of SDS-PAGE analysis between PEF and non-PEF control samples. No significant change in surface hydrophobicity after PEF treatment.	[95]
Lactoferrin	Intensity of 35 kV/cm, pulse width of 2 µs, and pulse frequency of 200 or 100 Hz.; flow rate of 60 mL/min.	No significant differences in surface hydrophobicity values between PEF- and non-PEF-treated lactoferrin. No significant change in surface hydrophobicity.	[24]
β-lactoglobulin	Intensity of 12.5 kV/cm with 40 µF of capacitance. 1–10 pulses, with 15 s between pulses.	PEF partially denatured β-lactoglobulin.	[31]
Whole milk	Intensity of 20 or 26 kV/cm for 34 µs, bipolar square wave pulses, pulse width of 20 µs for 34 μs.	The surface hydrophobicity of milk proteins increased with increased electric field intensity.	[97]
Sodium caseinate	10–150 V/cm for 5 s—2 h using a 60 Hz sine wave alternating current.	Moderate electric field altered the secondary structure of sodium caseinate and unfolded the protein molecules.	[33]
β-lactoglobulin	20 V/cm during holding and 80 V/cm during heating at a frequency of 20 kHz for 5–7 min.	Changes in the secondary structure of β-lactoglobulin.	[99]
Bovine serum albumin (BSA)	Strengths of 78, 150, 300, and 500 V/m for 3 h.	Low-intensity electric field changed the tertiary structure of BSA.	[96]

**Table 4:** Effects of pulsed electric field (PEF) on the structure of plant proteins.

Plant Protein	PEF Conditions	Structural Changes	References
Soy protein isolate (SPI)	0–40 kV/cm for 0–547 μs, 2 ms pulse width, and 500 pulse per second (pps) pulse frequency.	- PEF caused slight changes in the secondary structures. - PEF treatment caused denaturation and aggregation of SPI.	[29]
SPI	0 to 50 kV/cm, 40 μs pulse width, 1.0 kHz frequency, and 10 mL/min flow speed.	- PEF changed the vibration of polar groups and reduced the strength of hydrogen bonding, leading to a decrease in the β-turns and an increase in the antiparallel β-sheets.	[101]
Sunflower protein	10–150 V/cm for 5 s-2 h at 25–45 °C.	- Moderate electric field at 150 V for 20 s altered the secondary and tertiary structures of sunflower protein.	[33]
Canola protein	10 to 35 kV, pulse frequency of 600 Hz, and pulse width of 8 μs.	- PEF caused protein molecule aggregation. - PEF reduced β-turns and random coils and increased α-helices and β-sheets.	[69]
Pea protein isolate	5, 10, and 20 V/cm and frequencies of 50 Hz and 20 kHz.	- Moderate electric field treatment (50 Hz and 20 V/cm) unfolded the α-helix into a β-sheet structure. - Aromatic amino acids were exposed to the solvent.	[102]

**Table 5:** Effects of pulsed electric field (PEF) on the techno-functional properties of dairy proteins.

Dairy Protein	PEF Conditions	Changes in Protein Functionality	References
Raw milk	Intensity of 30 kV/cm, outlet temperature of 50 ± 1 °C; pulse number of 80 and 120 pulses, pulse width of 2 µs, and pulse frequency of 2 Hz.	Rennet coagulation time (RCT) higher than that of raw milk but lower than that of pasteurized milk.	[105]
Whey protein isolate (WPI)	15–22 V/cm heating phase and 4 to 8 V/cm holding phase, frequency of 25 kHz.	Moderate electric field treatment resulted in a weaker gel structure than conventional heat treatment.	[106]
β-lactoglobulin	20 V/cm during holding, 80 V/cm during heating, and frequency of 20 kHz.	At pH 7, moderate electric field and thermal treatment (up to 60 °C) had similar effects on the free SH group relativity. At higher temperatures, conventional heat-treated samples had higher free-SH-group relativity than moderate electric field-treated samples.	[99]
WPI	30–35 kV/cm, 19.2–211 µs, 30–75 °C.	Emulsions stabilized by PEF-treated and heat-treated (72 °C for 15 s) WPI showed similar droplet sizes and similar emulsifying properties. Increasing the duration of heat treatment to 10 min caused a significant increase in the droplet size of emulsions stabilized by heat-treated WPI. PEF-treated WPI showed lower gel strength than untreated samples. Increasing the duration of PEF further decreased the gel strength.	[95]
β-lactoglobulin	Intensity of 12.5 kV/cm with 40 µF of capacitance.	PEF improved the gelling rate of β-lactoglobulin (at 72 °C) when the number of pulses was less than six.	[31]
WPI	15 to 55 kV/cm, 2 to 8 and 50 to 90 °C.	The gelling properties of WPI increased when treated at 35 kV/cm but decreased after treatment at 45 kV/cm.	[107]

**Table 6:** Effects of pulsed electric field (PEF) on the techno-functional properties of plant proteins.

Plant Protein	PEF Conditions	Changes in Protein Functionality	References
Soy protein isolate (SPI)	0–40 kV/cm for 0–547 μs, 2 ms pulse width, and 500 pulse per second (pps) pulse frequency.	- PEF decreased the solubility and surface hydrophobicity.	[29]
Canola protein	10 to 35 kV, pulse frequency of 600 Hz, and pulse width of 8 μs.	- PEF treatment improved several functional properties of canola protein, including solubility, foaming, and emulsifying properties.	[69]
Sunflower protein	10–150 V/cm for 5 s–2 h at 25–45 °C.	- Moderate electric field treatment at 20 V reduced the interfacial tension at the sunflower protein solution/water interface.	[33]
Pea protein isolate	5, 10, and 20 V/cm and frequencies of 50 Hz and 20 kHz.	- Moderate electric field treatment (50 Hz and 20 V/cm) increased the surface hydrophobicity and improved the gelling properties of pea protein.	[102]

## Data Availability

Data is contained within the article.
